# Genome-wide survey of Gγ subunit gene family in eight Rosaceae and expression analysis of *PbrGGs* in pear (*Pyrus bretschneideri*)

**DOI:** 10.1186/s12870-021-03250-9

**Published:** 2021-10-15

**Authors:** Guodong Chen, Yang Li, Xin Qiao, Weike Duan, Cong Jin, Rui Cheng, Jizhong Wang

**Affiliations:** 1 College of Life Science and Food Engineering, Huaiyin Institute of Technology, Huai’an, 223003 China; 2 Center of Pear Engineering Technology Research, State Key Laboratory of Crop Genetics and Germplasm Enhancement, College of Horticulture, Nanjing Agricultural University, Nanjing, 210095 China; 3 Huai’an Key Laboratory for Facility Vegetables, Huaiyin Institute of Agricultural Sciences of Xuhuai Region of Jiangsu, Huai’an, 223001 China

**Keywords:** Gγ subunit, Pear, Expression pattern, Abiotic stress, Subcellular localization

## Abstract

**Background:**

Heterotrimeric G-proteins, composed of Gα, Gβ and Gγ subunits, are important signal transmitters, mediating the cellular response to multiple stimuli in animals and plants. The Gγ subunit is an essential component of the G-protein, providing appropriate functional specificity to the heterotrimer complex and has been well studied in many species. However, the evolutionary history, expression pattern and functional characteristics of Gγ subunits has not been explored in the Rosaceae, representing many important fruit crops.

**Results:**

In this study, 35 Gγ subunit genes were identified from the eight species belonging to the Rosaceae family. Based on the structural gene characteristics, conserved protein motifs and phylogenetic analysis of the Gγ subunit genes, the genes were classified into three clades. Purifying selection was shown to play an important role in the evolution of Gγ subunit genes, while a recent whole-genome duplication event was the principal force determining the expansion of the Gγ subunit gene family in the subfamily Maloideae. Gγ subunit genes exhibited diverse spatiotemporal expression patterns in Chinese white pear, including fruit, root, ovary and bud, and under abiotic stress conditions, the relative expression of Gγ subunit genes were up-regulated or down-regulated. In addition, seven of the Gγ subunit proteins in pear were located on the plasma membrane, in the cytoplasm or nucleus.

**Conclusion:**

Overall, this study of the Gγ subunit gene family in eight Rosaceae species provided useful information to better understand the evolution and expression of these genes and facilitated further exploration of their functions in these important crop plants.

**Supplementary Information:**

The online version contains supplementary material available at 10.1186/s12870-021-03250-9.

## Background

Heterotrimeric GTP-binding proteins (G-proteins) are critical for sensing external cues and transducing the signals into cells to participate in a variety of adaptive growth and developmental responses in plants and animals [1–3]. Such responses in plants include regulation of cell growth, cell differentiation, defense, stomatal movement and ion channels, and especially the perception of plant hormones and sugar signals [4]. The gamma (Gγ) subunit is one of the most important parts of the G-protein, binding tightly to the beta (Gβ) subunit and anchoring the Gαβγ trimer to switch the incoming signal on or off by separating from or binding to the alpha (Gα) subunit in plants [5–7]. In plants, the first γ-subunit homolog gene was cloned and characterized from *Arabidopsis* in 2000 [8], following which further γ-subunit homolog genes have been identified and cloned from other plant species. In humans, a total of 12 γ-subunit homolog genes have been reported, which have been shown to employ numerous combinations of their gene products to distinguish between various signals [9]. The number of γ-subunit homolog genes is three in *Arabidopsis* [2], four in tomato [10], five in rice [11] and 10 in soybean [12], and the cloning and functional analysis of γ-subunit homolog genes have also been well-studied in cucumber [7] and other plant species [13].

The structure of the animal Gγ subunit has been studied in detail [14]. The γ-subunit forms a coiled-coil structure with its β-subunit partner on the N-terminus of the γ-subunit domain, and there is a CAAX motif on the C-terminus of the γ-subunit domain [15]. In contrast, the structure of the γ-subunit in plants shows diversity and can be divided into three different structural subtypes, namely I, II and III [16]. Type I represents the canonical form of the Gγ subunit and is structurally similar to the Gγ subunits found in animal cells and their fungal counterparts [17]. For example, *Arabidopsis* Gγ1 and Gγ2 belong to type I and these proteins show similar molecular weights, a conserved domain for coil interaction with Gβ, and a conserved prenylation signal at the C-terminus [17]. Type II Gγ subunits are similar to type I Gγ subunits, possessing the N-terminal Gγ domain but lacking the C-terminal CAAX motif. Gγ subunits, such as RGG2 in rice and SlGGB1 in tomato, belong to type II Gγ subunits, although no members of the type II Gγ subunits members have been found in *Arabidopsis* [18]. The type III Gγ subunits, represented by GmGγ8, GmGγ9 and GmGγ10 in soybean and AGG3 in *Arabidopsis* [12, 19, 20], have been recently discovered and are novel, plant-specific proteins that possess unique features compared with all other Gγ subunits [21]. For example, these proteins are almost twice as large as the type I or type II Gγ subunits, and contain a modular structure, with a Gγ-like domain at its N-terminus, followed possibly by a transmembrane domain and a long cysteine-rich C-terminal region [17].

Gγ proteins are involved in the regulation of various signaling pathways affecting growth and development in plants. For example, studies have shown that AGG1 from *Arabidopsis* participates in auxin signaling with brassinosteroids [22]. The type II Gγ protein from *Brassica napus*, BnGG2, also participates in hormone signaling pathways and may be involved in plant defense systems against environmental stresses [23]. The transcript abundance of the RGG1 and RGG2 genes in rice were upregulated in response to NaCl, cold or heat stress, or to ABA exposure, which suggested that the Gγ subunit played a critical role, *via* cross talk, in signaling pathways for promoting stress tolerance in plants [24, 25]. In cucumber, transgenic cucumber plants (T_1_ generation) constitutively overexpressing *CsGG3.2* exhibited tolerance to chilling conditions and increased expression of the drought-tolerance CBF (C-repeat (CRT)/dehydration-responsive element (DRE)) genes and their regulon [7].

In tomato, researchers showed that type II Gγ subunits were involved in abscisic acid (ABA) and auxin signaling pathways during seed germination and fruit development, respectively [10]. Functional analysis of *Arabidopsis* type III AGG3 demonstrated its involvement in G-protein-mediated ABA signaling pathways [19], regulating organ size [19, 26]. In soybean, the type III Gγ proteins are involved during ABA-dependent inhibition of nodule formation and lateral root development in transgenic soybean hairy roots [27]. In addition, the type III Gγ proteins in rice, RGG3 (GS3), RGG4 (DEP1) and RGG5 (GGC2), are associated with important quantitative trait loci (QTLs) for regulating seed length and plant architecture variables, including semi-dwarfness, panicle number and panicle erectness. These Gγ proteins increased grain length individually or in combination with DEP1 [28, 29]. Similar results were obtained in heterologous expression systems or fungi, with overexpression of AGG3 in *Camelina sativa* resulting in increases in yield and heavy metal stress tolerance [30]. Furthermore, the Gγ subunit MGG1 from the phytopathogenic fungus *Magnoporthe oryzae* might act upstream of the cyclic AMP (cAMP) signaling pathway and may play critical roles in the regulation of conidiation, appressorium formation, mating and plant infection in the causal agent of rice blast, *M*. *oryzae* [31].

As noted previously, Gγ subunit proteins play an important role in signal transduction of plant growth and development, and the members and function of the Gγ subunit have been widely identified and investigated in various plants, but the members of the Gγ subunit gene family and their functions in the Rosaceae family have not been examined in detail. In other plant families, genome analysis, facilitated by the development of high-throughput sequencing technology, has been widely used to investigate the Gγ subunit gene family. Furthermore, pear and other Rosaceae fruit species are widely cultivated all over the word which are very popular for its nutritional and delicious. Recently, the genome of the pear (*Pyrus bretschneideri*) has been fully sequences and released, and genome sequences are also available for seven other Rosaceae species (apple, peach, strawberry, Japanese apricot, sweet cherry, black raspberry, and European pear). These data provide an opportunity to further analyze the Gγ subunit gene family in Rosaceae species. Therefore, in this study, we used bioinformatics and molecular biology techniques to identify and characterize the Gγ subunit gene members, the driving forces behind the evolution of these genes and their evolutionary history, expression patterns and subcellular localization in eight economically important fruit crop species in the Rosaceae. Our results identified a set of potential candidates Gγ subunit genes to carry out further investigations of the functions and signal transduction pathways mediated by Gγ subunit proteins in these important plants.

## Results

### The Gγ subunit gene family in the Rosaceae

**Fig. 1:**
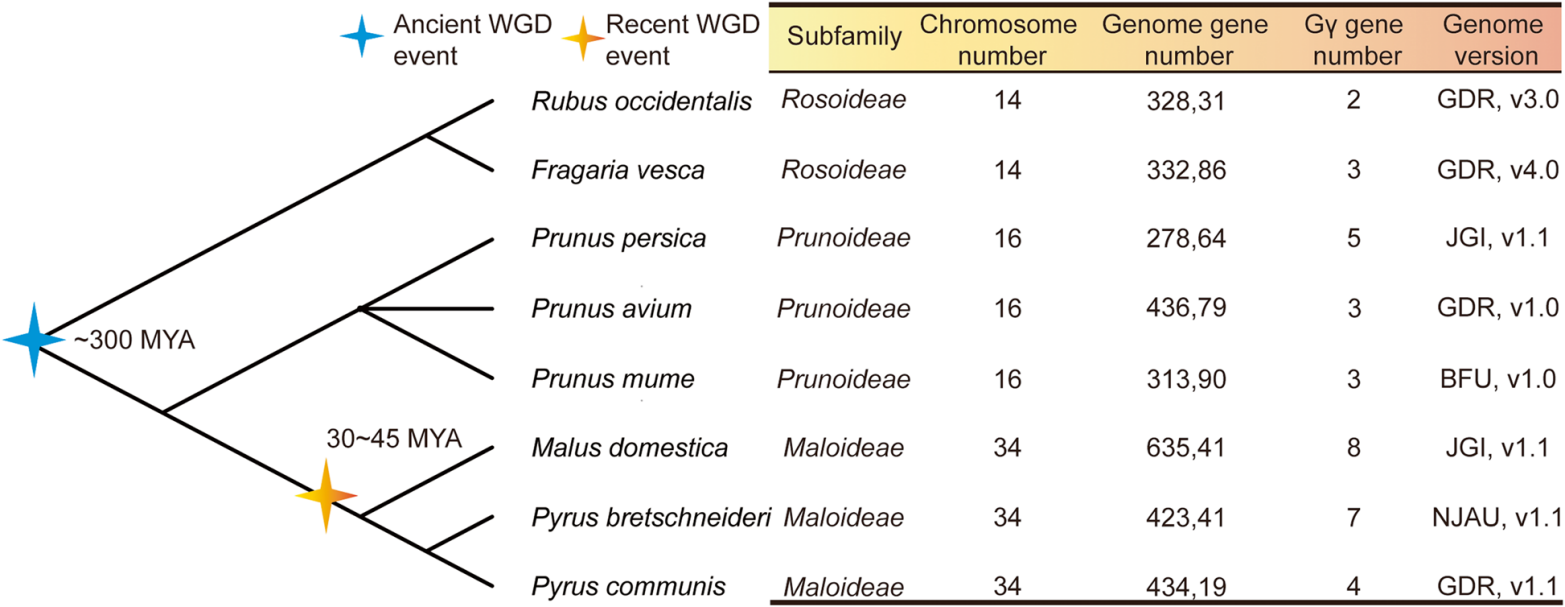
The evolutionary relationships and genome information of eight Rosaceae species. Blue and orange stars represent the divergent events in the eight species. The blue star indicates the occurrence of the ancient whole-genome duplication γ event (~300 mya) in all members of the Rosaceae; the orange star indicates the occurrence of the recent, lineage-specific WGD event (30~45 mya) in apple and pear. The data were downloaded from NCBI Taxonomy common tree (http://www.ncbi.nlm.nih.gov/Taxonomy/CommonTree/wwwcmt.cgi), and the phylogenetic tree was constructed using MEGA6 software

To identify the members of the Gγ subunit gene family in the Rosaceae, we searched the entire genome sequences of each of the eight Rosaceae species, using two strategies: one approach was to use a Hidden Markov Model (HMM) file of the Gγ subunit gene domain (PF00631.22) from Pfam to screen the genomes of the eight Rosaceae species, whereas the other was to use the *Arabidopsis* amino acid sequence of the Gγ subunit as queries with which to carry out BLASTP searches against the genome databases of each of the eight Rosaceae species. Thirty-nine candidate Gγ subunit genes were identified. Subsequently, redundant sequences, incomplete gene sequences and incomplete domain sequences were excluded by sequence alignment, with the completeness of the Gγ subunit gene domains being analyzed using Pfam and SMART. Ultimately, a total of 35 complete and nonredundant Gγ subunit genes were identified from the eight Rosaceae species, namely seven Gγ subunit genes in Chinese white pear, three in strawberry, eight in apple, four in European pear, three in sweet cherry, five in peach, two in black raspberry and three in Japanese apricot (Fig. 1).

Previous studies had shown that whole-genome duplication (WGD) events play a crucial role in the expansion and evolution of plant genes [32]. In the apple genome, two rounds of WGD events have been observed, a recent WGD (Ks -0.2) and the paleoduplication event corresponding to the γ triplication (Ks -1.6) [33, 34]; while in the pear genome, a recent WGD occurred 30-45 MYA (Ks 0.15-0.3) and an ancient WGD from a γ event occurred 140 MYA (Ks 1.5-1.8) [34]. However, since the divergence of the Rosoideae, Maloideae and Prunoideae subfamilies, the recent lineage-specific WGD event only occurred in the Maloideae (Fig. 1). There were more members of the Gγ subunit gene in members of the Maloideae than in both the Rosoideae and the Prunoideae combined. Therefore, we could infer that the recent WGD event played a crucial role in the expansion of the Gγ subunit gene family in the Maloideae.

### Sequence features of the Gγ subunit genes in the Rosaceae

Characteristics of the Gγ subunit protein were investigated using the Expasy server, in order to better understand the structures and functions of the Gγ subunit genes in the Rosaceae. The results showed that the lengths of the amino acid (aa) sequences ranged from 81 to 265 aa, with most of them being between 104 and 146 (Table 1). The protein molecular weights ranged from 9.10 to 24.06 kDa, and the isoelectric points (pI) varied from 4.25 to 10.02. The isoelectric point value of 68.57% of the Gγ subunit proteins was less than 7, which indicated that these proteins were rich in acidic amino acids. In addition, the grand average of hydropathicity index (GRAVY) of each protein was calculated, reflecting their hydrophobicity or hydrophilicity, by calculating the sum of the hydropathy values of all the amino acids divided by the sequence length [35]. The results showed that the GRAVY value of 85.71% of the Gγ subunit proteins was less than 0, indicating that these proteins were hydrophilic. Finally, the aliphatic index of the Gγ subunit proteins in the eight Rosaceae species was analyzed, with the results showing that most of them were quite variable. The aliphatic indices of the Gγ subunit proteins ranged from 42.95 to 112.42, which indicated that these Gγ subunit proteins were thermostable (Table 1).

**Table 1:** Characteristics of the Gγ subunit proteins in the eight Rosaceae species

Gene name	Subfamily	protein length (aa)	Protein molecular weight (Da)	PI	GRAVY	Formula	Aliphatic index
FvH4_1g08850.1	III	225	24063.86	9.06	0.089	C_1070_H_1720_N_310_O_303_S_9_	98.4
FvH4_4g01800.1	I	81	9100.18	4.93	-0.774	C_386_H_638_N_114_O_133_S_3_	77.16
FvH4_2g30640.1	II	125	13504.09	5.9	-0.52	C_584_H_927_N_175_O_186_S_4_	73.44
MD13G1202200	I	105	12019.55	5.38	-0.849	C_523_H_830_N_150_O_165_S_5_	68.86
MD15G1110600	II	129	13889.39	5.46	-0.549	C_607_H_946_N_178_O_193_S_2_	71.86
MD15G1218900	III	211	23778.15	8.68	-0.256	C_994_H_1574_N_300_O_282_S_48_	44.83
MD03G1256500	I	112	12197.54	4.29	-0.378	C_525_H_836_N_148_O_178_S_4_	87.86
MD16G1202400	I	105	12048.59	5.36	-0.789	C_524_H_833_N_151_O_165_S_5_	72.57
MD08G1133500	II	130	14162.69	5.16	-0.647	C_617_H_961_N_181_O_197_S_3_	69.85
MD11G1277200	I	115	12511.96	4.27	-0.319	C_544_H_862_N_150_O_180_S_4_	92.35
MD02G1093600	III	227	25213.02	8.54	-0.078	C_1046_H_1669_N_311_O_301_S_56_	48.55
Pav_sc0000129.1_g1210.1.mk	III	218	23623.71	9.48	0.302	C_1082_H_1696_N_294_O_282_S_9_	97.52
Pav_sc0000983.1_g380.1.mk	I	113	12246.75	4.72	-0.275	C_530_H_851_N_153_O_172_S_4_	92.39
Pav_sc0003562.1_g080.1.mk	II	143	15719.31	5.78	-0.774	C_681_H_1058_N_204_O_220_S_3_	62.8
Pm004925	II	142	15470.98	5.78	-0.824	C_664_H_1042_N_202_O_220_S_3_	62.54
Pm020240	I	112	12257.64	4.43	-0.362	C_526_H_844_N_152_O_177_S_4_	90.54
Pm027095	III	206	22507.71	9.42	0.219	C_1014_H_1623_N_279_O_267_S_16_	94.08
Prupe.2G330000.1.p	II	146	15970.54	5.66	-0.793	C_687_H_1079_N_205_O_229_S_3_	63.56
Prupe.8G233500.1.p	I	113	12286.64	4.43	-0.384	C_526_H_843_N_153_O_178_S_4_	88.05
Prupe.1G462900.1.p	II	146	15859.4	5.78	-0.787	C_680_H_1070_N_206_O_227_S_3_	62.88
Prupe.1G024000.1.p	I	105	11993.51	5.41	-0.842	C_520_H_828_N_152_O_164_S_5_	68.86
Prupe.7G197000.1	III	234	26038.81	8.6	-0.174	C_1074_H_1704_N_324_O_314_S_58_	42.95
Pbr012894.1 (PbrGG1)	I	114	12512.87	4.25	-0.41	C_541_H_853_N_151_O_182_S_4_	86.32
Pbr014702.1 (PbrGG2)	I	105	12019.55	5.38	-0.849	C_523_H_830_N_150_O_165_S_5_	68.86
Pbr018868.1 (PbrGG3)	I	129	14720.6	5.64	-0.769	C_635_H_1024_N_186_O_204_S_6_	72.71
Pbr002651.1 (PbrGG4)	II	132	14027.51	5.26	-0.505	C_613_H_952_N_180_O_195_S_2_	73.94
Pbr021253.1 (PbrGG5)	II	129	13959.55	4.99	-0.487	C_608_H_952_N_176_O_194_S_4_	74.88
Pbr027751.1 (PbrGG6)	III	204	22757.6	10.02	-0.013	C_1035_H_1641_N_297_O_270_S_6_	95.59
Pbr037429.2 (PbrGG7)	III	209	23449.94	8.66	-0.187	C_976_H_1562_N_294_O_277_S_50_	49
pycom02g07380	III	265	29871.49	8.59	0.016	C_1292_H_2000_N_362_O_348_S_53_	57.02
pycom03g20330	I	114	12624.01	4.34	-0.472	C_546_H_862_N_154_O_182_S_4_	84.56
pycom11g24520	I	114	12437.89	4.43	-0.392	C_540_H_856_N_152_O_177_S_4_	87.19
pycom15g19410	III	211	23694.15	8.7	-0.245	C_983_H_1574_N_300_O_281_S_50_	46.68
Ro02_G04020	I	104	11699.13	4.35	-0.332	C_508_H_814_N_140_O_168_S_4_	97.5
Ro01_G03005	III	248	27144.88	9.2	0.298	C_1232_H_1975_N_343_O_329_S_9_	112.42

### Classification, gene structure and conserved protein motif analysis of Gγ subunits in members of the Rosaceae

**Fig. 2:**
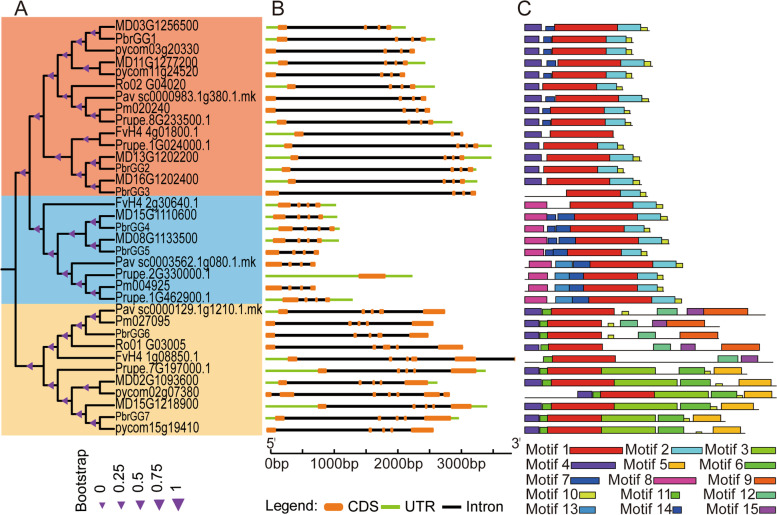
Phylogenetic tree, structural gene features and conserved protein motifs of the Gγ subunit genes in the eight Rosaceae species. **A** The phylogenetic tree was generated using the amino acid sequences encoded by each of 35 Gγ subunit genes with the Neighbor-Joining method and 1,000 bootstrap replicates. Different colors in the branches indicated the different subunit type. **B** Gene structural analysis, the orange boxes, black lines and green boxes in the gene structural diagram represent coding sequences (CDS), introns and untranslated regions (UTRs), respectively; gene models are drawn to scale as indicated on the x-axis. **C** Conserved motif analysis: fifteen distinct motifs (motifs 1 to 15) were identified with the MEME tool and the representation of each motif was illustrated with a different color. The lengths and positions of the colored blocks correspond to the lengths and positions of the motifs in the individual protein sequences, respectively

To classify the Gγ subunit genes identified in members of the Rosaceae, in order to investigate their evolutionary relationships, a phylogenetic tree was constructed, based on the protein sequences, using the Neighbor-Joining (NJ) method. Based on the phylogenetic tree, the Gγ subunit genes in eight rosaceous species were divided into three types (I, II and III) (Fig. 2A). Subsequently, the structural diversity of Gγ subunit genes was analyzed by comparing the numbers of introns and exons. The results showed that there was no significant difference in the number of exons in each type, with the number of exons in types I and II being the same, namely four exons, except for *FvH4_ 4g01800.1* (three) and *Prupe.2G330000.1*(one) (Fig. 2B). However, the number of exons in type III was slightly higher than that in types I and II, namely five exons, except for *FvH4 1g08850.1* (six) and *pycom02g07380* (seven).

In order to further investigate the diversity of Gγ subunit gene structure and function in each type, we performed conserved motif analysis on the Gγ subunit protein sequences in the eight Rosaceae species, using the MEME program. The results showed that 15 consensus motifs were identified, namely motif 1–15 (Fig. 2C). The number of motifs present in the Gγ subunit protein sequences of each type was variable, ranging from two to seven. Among them, the members of type I had the fewest conserved motifs, which ranged from two to five, with motifs 1 and 2 being present in all the Gγ subunit proteins of type I. Members of clade II contained an intermediate number of conserved motifs, which ranged from four to six, with motifs 1, 2, 8 and 10 being universally distributed in the Gγ subunit protein family of type II. Type III contained the highest number of conserved motifs, with the largest number in a single protein being seven, with motifs 1 and 11 being present in all the Gγ subunit proteins of type III. Therefore, motif 1 was determined to be the conserved domain of the Gγ subunit protein based on the above results from the conserved motif analysis, because it was identified in all 35 members of the Gγ subunit protein family of the Rosaceae. The phylogenetic relationship, structural diversity and conserved motif analysis of the Gγ subunit proteins showed that the conservation and specificity of the number of conserved motifs and exons in different subunit types may have originated *via* different evolutionary paths and may play varying functions in different organs.

### Evolutionary expansion of gene number and syntenic analysis of Gγ subunit genes in the Rosaceae

**Fig. 3:**
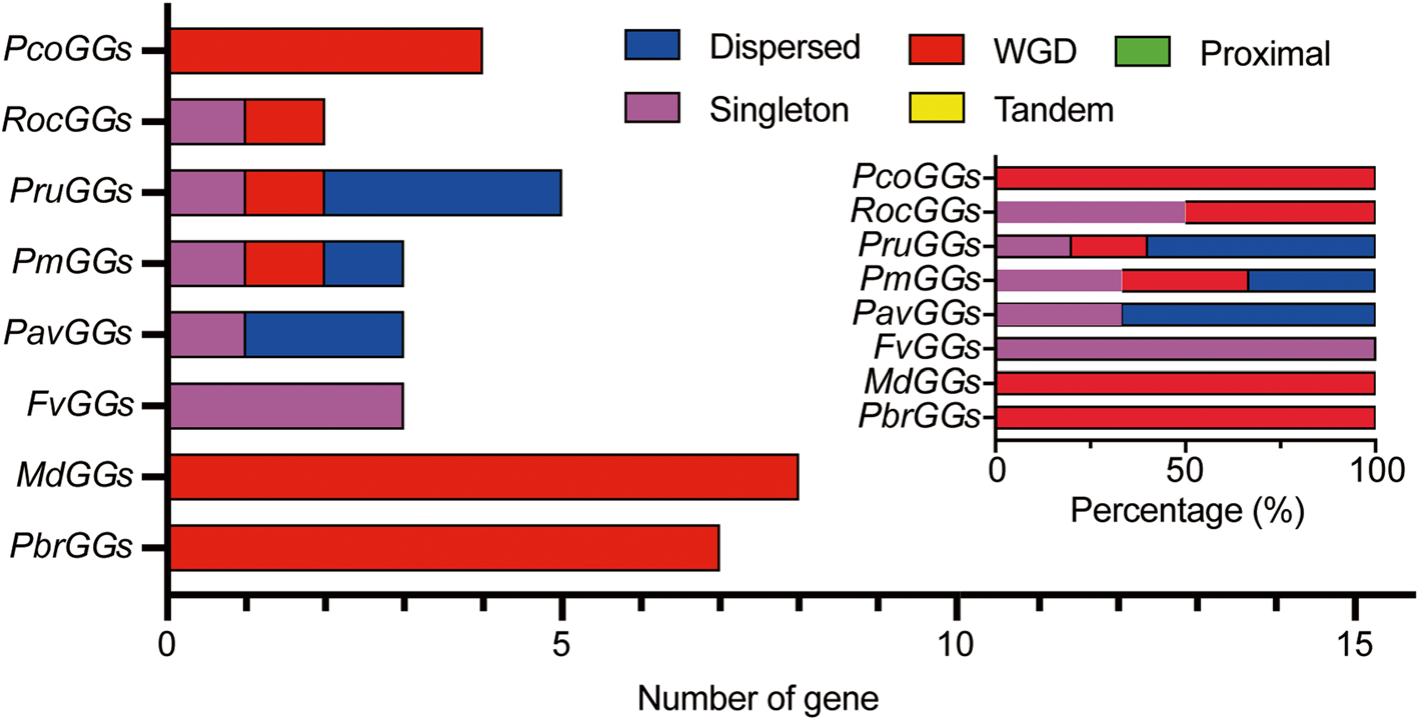
The number of Gγ subunit gene pairs from different origins in the eight Rosaceae species. The number of different modes of duplicated Gγ subunit gene pairs was determined by MCScanX and presented by GraphPad Prism 8 software. The x-axis in the lower histogram indicates the number of Gγ subunit genes. The x-axis of the upper-right histogram indicates the percentage frequency of each duplicated event. The y-axis of both histograms indicates the different species. Different color bars represent different duplication events

Gene duplication events contribute to the expansion of protein-coding gene families, consisting of five classes of gene duplication events, namely WGD (whole-genome duplication), dispersed, tandem, proximal and singleton duplications. To explore the evolutionary origins of the members of the Gγ subunit gene family in the Rosaceae, the duplication events occurring during evolution of the Gγ subunit gene family in the eight species of the Rosaceae were analyzed. Overall, three of the five classes of gene duplication events (WGD, singleton and dispersed) were identified in evolution of the Gγ subunit gene family in the Rosaceae species, with 20%, 62.9% and 17.1% of the Gγ subunit genes in the Rosaceae being duplicated and retained as a result of a singleton event, a WGD event, or a dispersed event, respectively (Fig. 3). More specifically, a WGD event was found to be responsible for expansion of only the Gγ subunit gene family in Chinese white pear, apple and European pear, which infers that the WGD event plays an important role in the expansion and evolution of the Gγ subunit gene family in only the Maloideae subfamily. In strawberry, only a singleton event was identified as being responsible for the expansion of the Gγ subunit gene family. Furthermore, 20%, 33.3%, 50% and 33.3% of the Gγ subunit genes were duplicated and retained from singleton events in peach, Japanese apricot, black raspberry and sweet cherry, respectively; 20, 33.3 and 50% of the Gγ subunit genes were duplicated and retained from a WGD event in peach, Japanese apricot, and black raspberry, respectively; and 60, 33.3 and 66.7% Gγ subunit genes were duplicated and retained from dispersed events in peach, Japanese apricot and sweet cherry, respectively (Table S1).

**Fig. 4:**
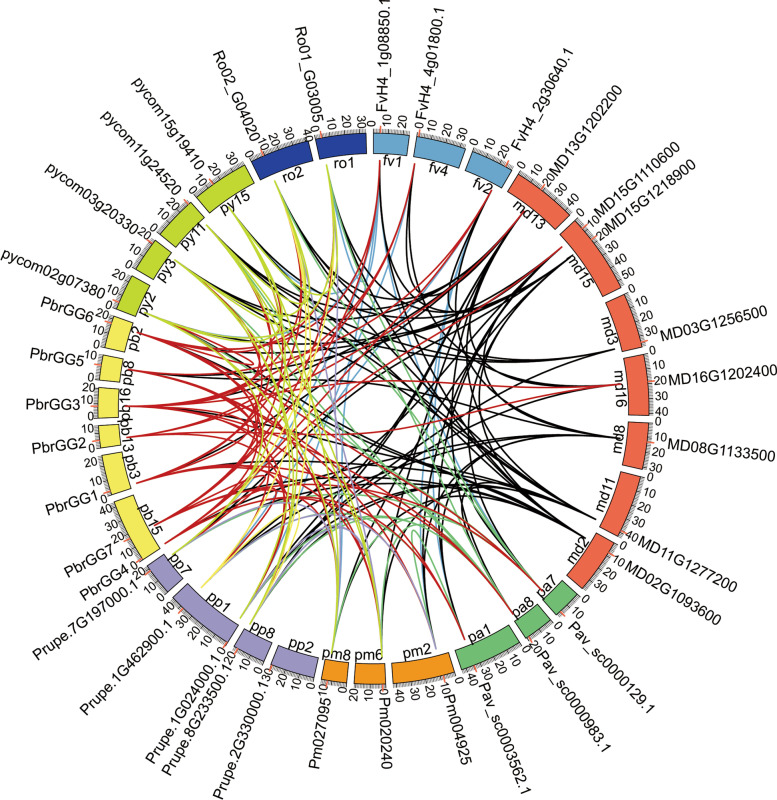
Distribution and collinearity of Gγ subunit genes in the eight Rosaceae species. The circular forms of the chromosomes of different species are marked in different colors. The red lines around the circumference of the circle mark the gene positions. The lines in different colors inside the circle represent the collinearity relationships among the genes from the eight Rosaceae species

To investigate the evolutionary roles and to better understand the diversity of the Gγ subunit gene family, we analyzed the syntenic relationships among the Gγ subunit genes of the eight Rosaceae species. In this study, each of the 35 Gγ subunit genes could be anchored to the chromosomes of one of the rosaceous species. Seven Gγ subunit genes were mapped onto six of the 17 chromosomes in Chinese white pear, and four Gγ subunit genes were assigned to Chr2, Chr3, Chr11, and Chr15 in European pear. Two Gγ subunit genes were located on two of the six chromosomes in black raspberry, and three Gγ subunit genes were distributed on Chr1, Chr2 and Chr4 in strawberry. Eight Gγ subunit genes were located to seven of the 17 chromosomes in apple, whereas three Gγ subunit genes were located on Chr1, Chr7 and Chr8 in sweet cherry. Three Gγ subunit genes were distributed on three of the eight chromosomes of Japanese apricot, while five Gγ subunit genes were located on Chr1, Chr2, Chr7 and Chr8 in peach (Fig. 4).

Synteny and collinearity are traditionally determined by searching for both inter- and intragenomic pairwise conservation blocks, and the maximum synteny was observed in apple, which contained eight Gγ subunit genes that were syntenic with 34 Gγ subunit genes among the eight Rosaceae species. In Chinese white pear, we also found that seven Gγ subunit genes were syntenic with 55 Gγ subunit genes among the eight Rosaceae species. In addition, 39, 33, 32, 27, 22 and 21 gene pairs showing collinear relationships were obtained in European pear, peach, sweet cherry, Japanese apricot, strawberry and black raspberry, respectively (Fig. 4).

### Ks value and Ka/Ks ratio analysis of Gγ subunit genes in the Rosaceae

**Fig. 5:**
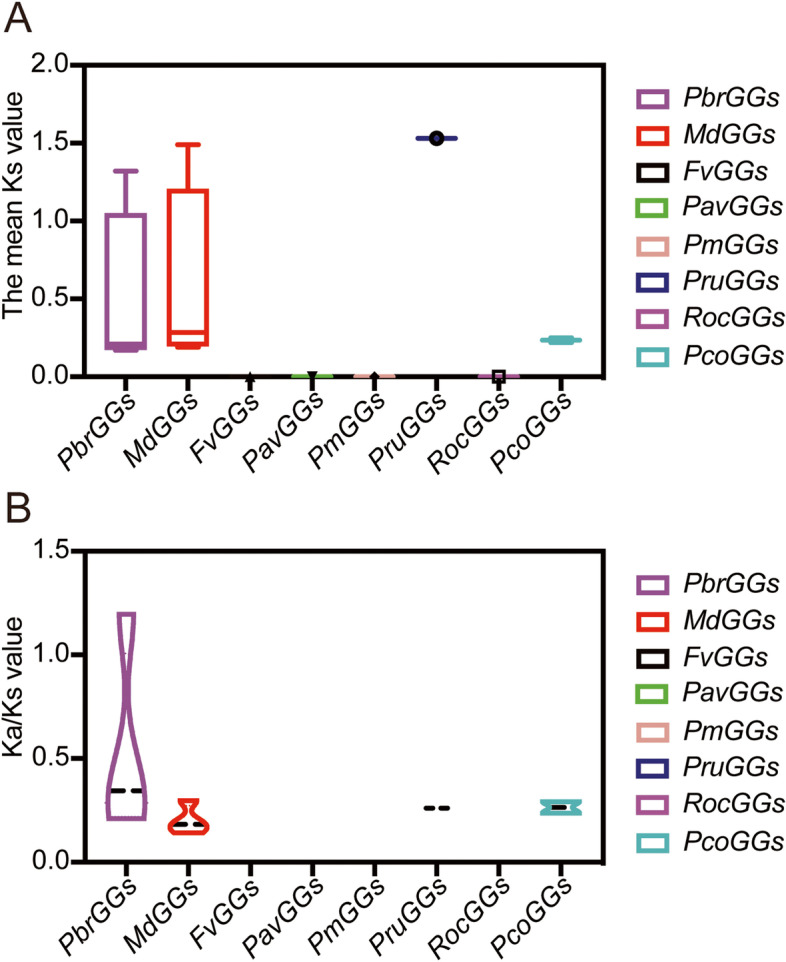
Distribution of the mean Ks and Ka/Ks values of Gγ subunit genes in the eight Rosaceae species. **A** The mean Ks values represent the times of Gγ subunit gene divergence in the eight Rosaceae species. **B** Ka/Ks ratios represent the driving forces behind duplicated Gγ subunit gene evolution in the eight Rosaceae species

The Ks value is commonly used to estimate the approximate occurrence dates of whole-genome duplication or segmental duplication events. According to previous research, two WGD events occurred in the Chinese white pear and apple genomes, one of them being an ancient WGD event (Ks~1.5–1.8 in pear, and Ks~1.6 in apple) which happened about ~140 mya, whereas the other was the recent WGD event (Ks~0.15–0.3 in pear, and Ks~0.2 in apple), which occurred about 30–45 mya [34]. To estimate the evolutionary dates of WGD events, the mean Ks of the Gγ subunit duplicated gene pairs on each side flanking region were analyzed in the Rosaceae species. The results showed that the mean Ks values for the Gγ subunit gene pairs ranged from 0.17 to 1.53 (Fig. 5A and Table S2). We further determined that the WGD event for the Chinese white pear gene pair *PbrGG4* and *PbrGG5* (Ks~0.23), the apple gene pairs *MD02G1093600* and *MD15G1218900* (Ks~0.17) and *MD13G1202200* and *MD16G1202400* (Ks~0.18) may have arisen from the recent WGD event, taking place about ~ 30–45 mya. Some duplicated gene pairs possessed similar but higher Ks values, such as *PbrGG3* and *PbrGG1* (Ks~1.9), indicating that these duplications may have been derived from the same, ancient WGD event, which occurred approximately ~140 mya. In addition, one duplicated apple gene pair (*MD11G1277200* and *MD13G1202200*) had an even higher Ks value (Ks ~ 2.1), suggesting that they might have resulted from an even more ancient duplication event. Furthermore, orthologous pairs and divergence time of Gγ subunit genes among the eight Rosaceae species were analyzed by analyzing syntenic relationships. The results showed that the number of orthologous gene pairs identified between Chinese white pear and the other seven Rosaceae species, namely strawberry, apple, sweet cherry, Japanese apricot, peach, European pear and black raspberry was six, fourteen, six, six, eight, eight and four, respectively (Table S3). The average Ks values of the Gγ subunit orthologs between Chinese white pear and strawberry, apple, sweet cherry, Japanese apricot, peach, European pear and black raspberry ranged from 0.01 to 2.31 (Table S3). The divergence time of the Gγ subunit genes for Chinese white pear and European pear ranged from 0.47 to 71.95 mya, moreover, the divergence time for Chinese white pear and the other six Rosaceae species was between 1.93 and 77.00 mya (Table S3). Among them, the Gγ subunit orthologs between Chinese white pear and apple had the lowest average Ks value (Ks ~ 0.51), indicating that the Gγ subunit gene evolutionary distance between these two species was closest among the eight Rosaceae species, whereas the Gγ subunit ortholog gene pairs between Chinese white pear and black raspberry exhibited the highest average Ka value (Ks ~ 0.81) (Table S3), signifying the most distant evolutionary distance.

To further explore which selection process drove the evolution of the Gγ subunit gene family, the Ka/Ks values of the paralogous Gγ subunit genes between the inter- and intragenomic pairwise blocks were analyzed in the eight Rosaceae species. Previous studies had shown that a Ka/Ks value greater than 1 indicates Darwinian (positive) selection, whereas a Ka/Ks value equal to 1 indicates neutral evolution, and a Ka/Ks value less than 1 indicates purifying (negative) selection [36]. Purifying selection is a process that can eliminate some disadvantageous mutations, whereas Darwinian selection can accumulate some new advantageous mutations and spread them throughout future generations. In the present study, the Ka/Ks value of most of the Gγ subunit orthologous gene pairs was less than 1, indicating that purifying selection was the main factor driving the evolution of the Gγ subunit gene family in the Rosaceae (Fig. 5B, Table S3). Surprisingly, two duplicated gene pairs (*PbrGG6* and *pycom02g07380*, *PbrGG7* and *PbrGG6*) showed higher Ka/Ks values (>1), suggesting that they might have undergone Darwinian selection (Fig. 5B, Table S3).

### Expression pattern analysis of *PbrGG* genes in different organs

**Fig. 6:**
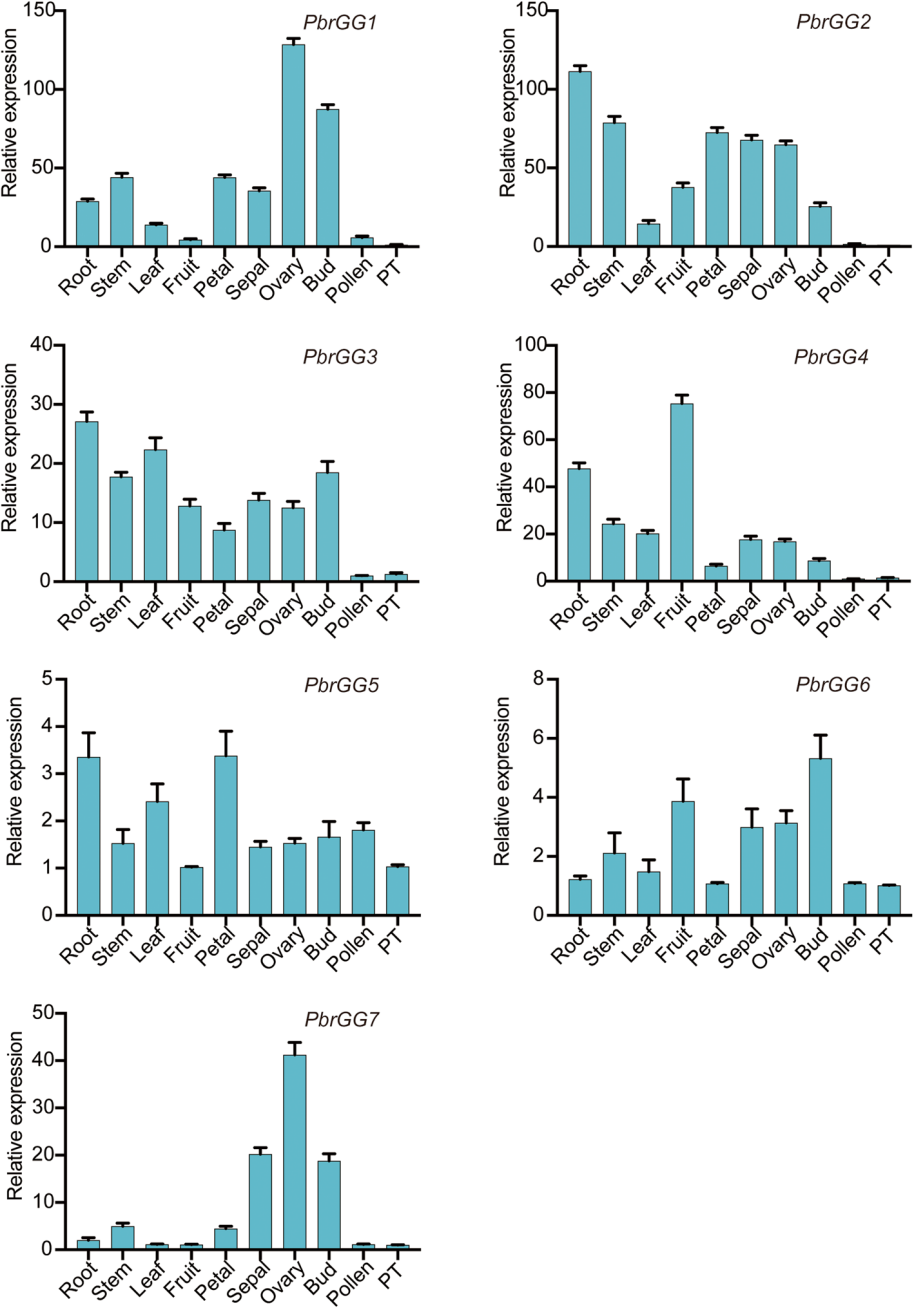
Expression analysis of seven *PbrGG* genes in different tissues. The relative transcript abundance levels of *PbrGG* genes were assessed by qPCR in various tissues of Chinese white pear. Total RNA was extracted from the root, leaf, stem, petal, bud, sepal, fruit, ovary, pollen and pollen tubes. For each gene, the relative expression levels were obtained by normalization with pear *Actin*. Data are shown as mean ± standard deviation

To explore the specific expression patterns and possible functional characteristics in various organs, seven *PbrGG* genes from Chinese white pear were chosen as candidate genes with which to establish a real-time quantitative PCR (qPCR) study to analyze the relative expression levels in root, stem, leaf, fruit, petal, sepal, ovary, bud, pollen and pollen tube of Chinese white pear. The results showed that the seven *PbrGG* genes exhibited a diversity of expression levels in different pear organs, suggesting that Gγ subunit genes may perform specific functions in different organs, participating in the corresponding metabolic processes. Overall, most of Gγ subunit genes were preferentially expressed in root, fruit, petal, ovary and bud tissues, indicating that Gγ subunit genes may play an important role in the growth and development of these organs. Specifically, *PbrGG4* was expressed at high levels in the fruit and root, but relatively weakly in the stem and the leaf, whereas *PbrGG1* and *PbrGG7* were highly expressed in the ovary, in addition to their expression in sepal and bud. The expression levels of *PbrGG2* in root, stem, petal, sepal and ovary were significantly higher than those in leaf, fruit and bud whereas *PbrGG3* expression showed preferential upregulation in the root, stem, leaf and bud whereas *PbrGG6* showed preferential expression in the fruit and the bud, with the expression levels of *PbrGG5* being upregulated not only in the root, leaf and petal but also in the pollen and the stem (Fig. 6).

### Expression patterns of *PbrGG* genes under abiotic stress

**Fig. 7:**
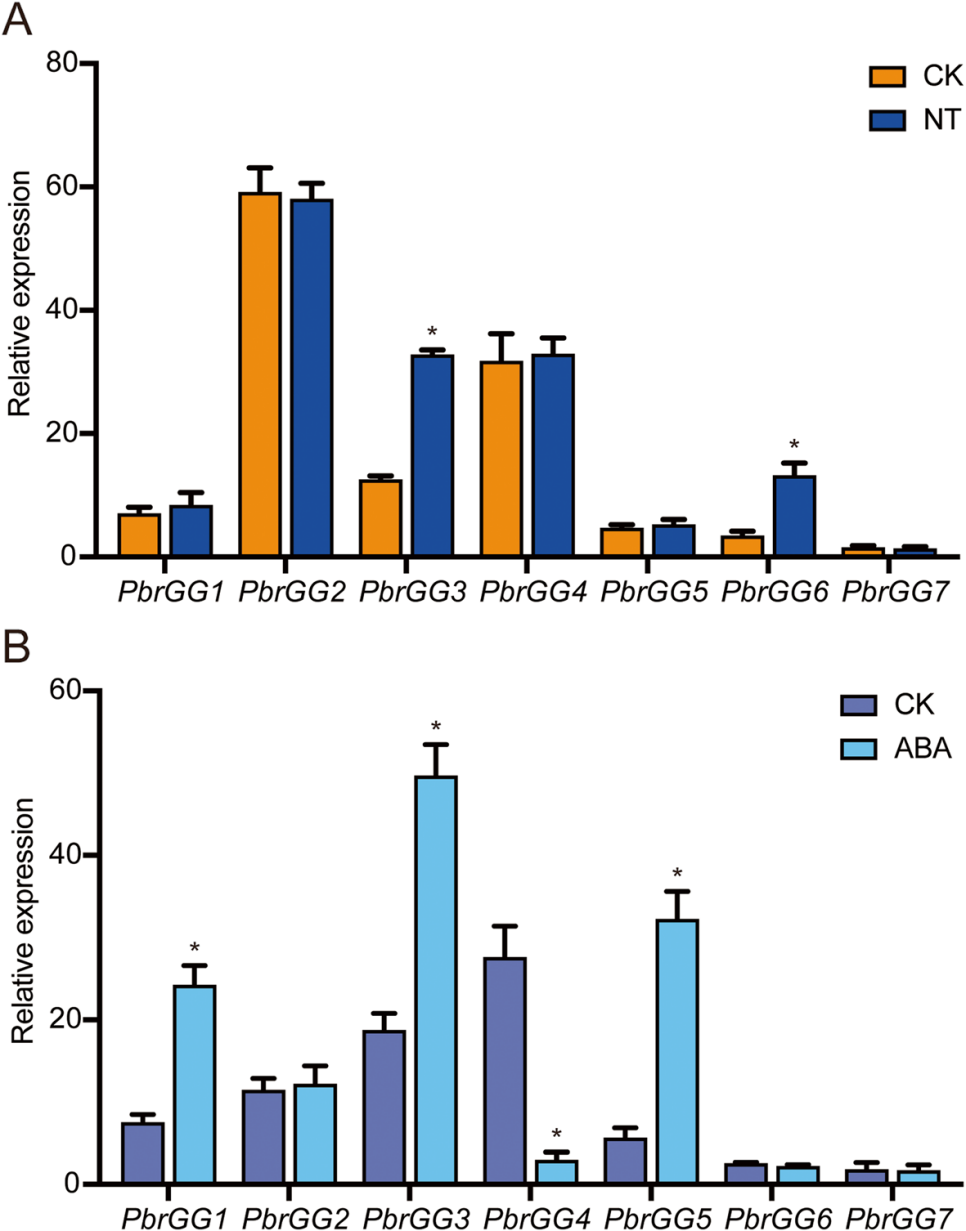
Expression patterns of *PbrGG* genes under abiotic stress. The relative expression levels of *PbrGG* genes were analyzed by qRT-PCR, and the samples were harvested from roots after N-deficiency treatment for five days; and the samples were harvested from leaves after ABA treatment for 6 h. For each gene, the relative expression levels were acquired by normalization to that of pear *Actin*. The error bars indicate standard deviations. Asterisks indicate a significant difference (*P < 0.05) compared with CK after abiotic stress treatment. Data are expressed as mean ± standard deviation

To further explore the potential functions of *PbrGGs* in response to various abiotic stress, pear seedlings were treated with nitrogen deficiency (N-deficiency) and ABA, and the relative expression levels of these genes were checked by qRT-PCR. The results showed that exposure to N-deficiency stress caused the relative expression levels of *PbrGG3*, and *PbrGG6* to increase by ~2.6 and ~3.8-folds in roots after five days as compared to CK, respectively; while *PbrGG1*, *PbrGG2*, *PbrGG4*, *PbrGG5*, and *PbrGG7* showed no significant changes in root under N-deficiency stress (Fig. 7A). Furthermore, in response to exogenous ABA treatment, each *PbrGG* genes showed a differential expression pattern. For example, *PbrGG1*, *PbrGG3* and *PbrGG5* were mainly up-regulated in leaves under ABA treatments (Fig. 7B). The expression levels of *PbrGG4* were significantly down-regulated after ABA treatment (Fig. 7B). However, the expression levels of *PbrGG2*, *PbrGG6* and *PbrGG7* remained unchanged after ABA treatment (Fig. 7B). These results suggest that the expression levels of *PbrGG* genes were induced by N-deficiency or ABA treatment and therefor may be involved in regulation of these biological processes responses related to it.

### Subcellular localization of PbrGG proteins in Chinese white pear

**Fig. 8:**
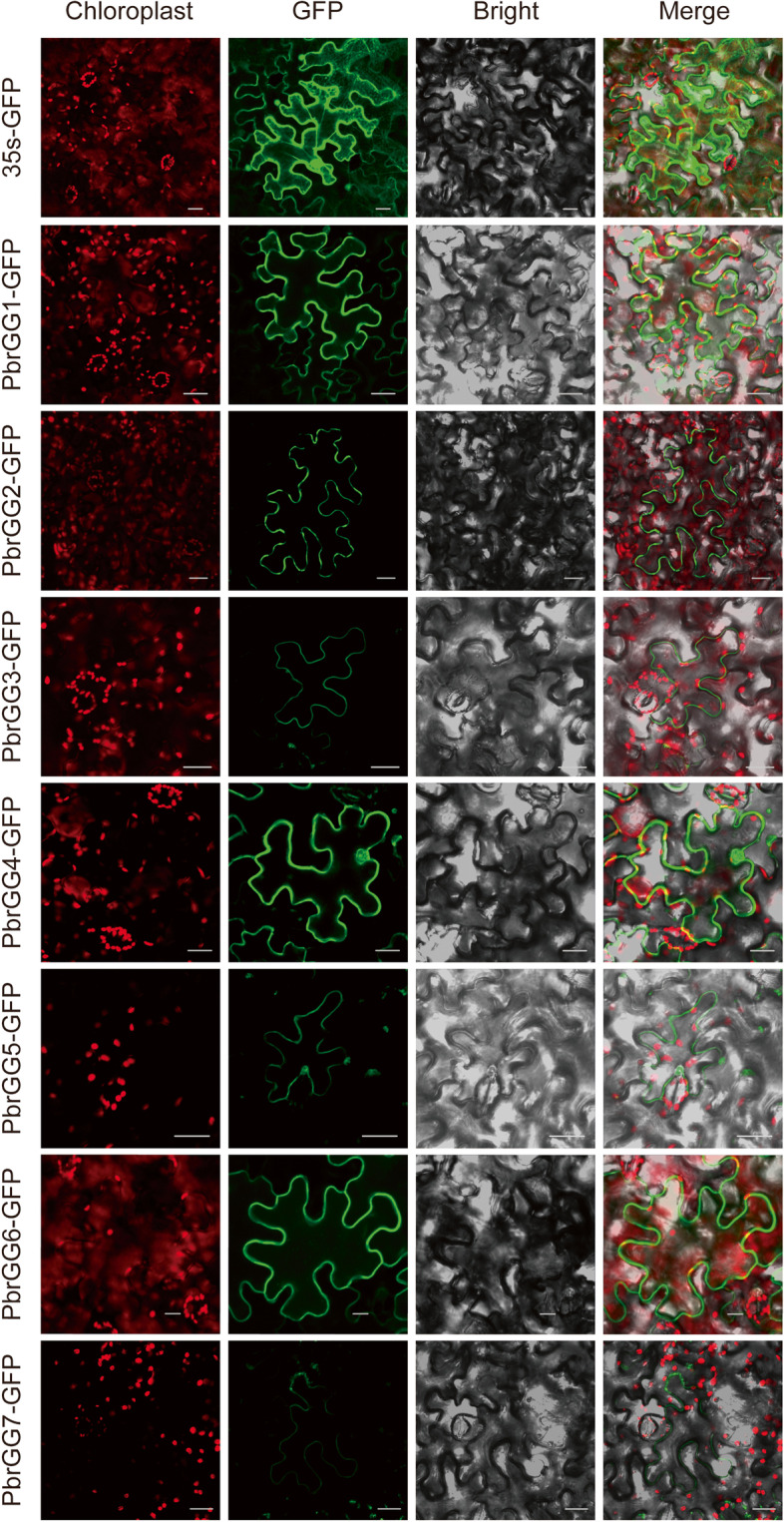
Subcellular localization of three PbrGG proteins. Seven PbrGG-GFP fusion proteins, with 35s-GFP as the control, were expressed transiently in tobacco leaves in an independent manner, and the results were visualized by laser confocal microscopy. The merged images include the chloroplast autofluorescence channel (first panels) and the green fluorescence channel (second panels). The corresponding brightfield images are shown in the third panels. Bar=20 μm

To further investigate the functions of the Gγ subunit genes in members of the Rosaceae, seven *PbrGG* genes were chosen as candidate genes with which to explore subcellular localization. Based on the presence of targeted sequences in the protein, the predictive results from the WoLF PSORT software showed that most of the Gγ subunit proteins were predicted to be located on the plasma membrane, in the nucleus and/or cytoplasm (Table S4). Seven Gγ subunit genes from different types were then selected to test the results of subcellular localization prediction experimentally, using transient expression assays of fusion proteins between the Gγ subunit and the reporter green fluorescent protein (GFP). The recombinant plasmid of 35s::*PbrGG*-*GFP* and control plasmid of 35s-*GFP* were transformed into *Nicotiana benthamiana* leaves by the *Agrobacterium*-mediated method. The results showed that the experiment data are basically identical with the WoLF PSORT software predicted ones. Using laser confocal microscopy, the green fluorescence was seen to be distributed across the entire cell when the control plasmid was used to transform the *Nicotiana benthamiana* leaves (Fig. 8). For tobacco cells transformed with the recombinant plasmids, however, the green fluorescence of *PbrGG1*-*GFP* from type I was observed on the plasma membrane and in the cytoplasm while that of *PbrGG2*-*GFP* and *PbrGG3*-*GFP* were observed only on plasma membrane. *PbrGG4*-*GFP* and *PbrGG5*-*GFP* from type II were observed on the plasma membrane and in the nucleus, whereas the fusion protein of *PbrGG6*-*GFP* and *PbrGG7*-*GFP* showed green fluorescence exclusively on the plasma membrane (Fig. 8). Therefore, these results indicated that Gγ subunit protein may play an important biological role in particular on the plasma membrane, as well as in the nucleus or cytoplasm depending on the identity of the subunit protein.

## Discussion

The Gγ subunit proteins have been implicated in growth and development processes, including the regulation of seed and organ size, and in signal transduction, individually or in combination with Gαβ. As an important part of G-protein, some members of the Gγ subunit protein family have been widely studied in various plants, such as *Arabidopsis* [37], rice [11], tomato [10] and cucumber [7]. However, there has been little research into annotation of the Gγ subunit proteins in members of the Rosaceae. In the current study, a total of 35 Gγ subunit genes were identified in eight rosaceous species, with the Gγ subunit gene family in Chinese white pear, European pear and apple having more members than those in strawberry, sweet cherry, peach, black raspberry or Japanese apricot. Furthermore, the number of Gγ subunit genes in Chinese white pear, European pear and apple was much higher than that reported in the model plant *Arabidopsis* [2]. In Fig. 1, we showed that the subfamily Maloideae had undergone both an ancient WGD event and a recent WGD event whereas only one ancient WGD event had occurred in species from the other two subfamilies [34]. These results indicated that the number of Gγ subunit genes had been expanded on a small scale by recent WGD events, resulting in an increase in the numbers of Gγ subunit genes in the Maloideae. Furthermore, gene duplication analysis showed that the expansion of the Gγ subunit genes in Chinese white pear, European pear and apple was due mainly to WGD events, while singleton and dispersed duplication events being identified as the major driving force for expansion of the Gγ subunit genes in the other Rosaceae species. The Ka/Ks ratios of all paralogous Gγ subunit pairs were less than 1, suggesting that the Gγ subunit genes underwent negative selection during the evolutionary process.

The plant Gγ subunit proteins are clearly distinguished into three different types based on their structural characteristics: type I (the classic one, resembling the subunits in animals), type II (lacking the isoprenylation motif) and type III (with a long tail) [10, 16, 38]. To classify and further explore the structural diversity of the Gγ subunit genes in Rosaceae, a phylogenetic tree was constructed, as well as analyses based on gene features, gene structures and protein conserved motifs were carried out in the current study. The results from the phylogenetic tree construct showed that all Gγ subunit genes were classified into three clades or types in the Rosaceae, namely I, II and III, a finding which is consistent with the classification from previous reports [16]. Furthermore, the results of gene structure and conserved protein motif analyses also supported the classification of the Gγ subunit genes in the Rosaceae, except for the loss of introns/exons or motifs in some Gγ subunit genes, with the characteristics of the Gγ subunit genes being similar within each type and varying among different types. This is consistent with findings from previous studies, among the three *Arabidopsis* Gγ subunit genes, AGG1 and AGG2, belonging to the same type, and strongly resembling the canonical mammalian Gγ subunit type I [8, 11, 39]. The type III Gγ subunit proteins are almost twice the size of the type I or type II Gγ subunits, with the number of amino acids in the type III Gγ subunit in the range 204–265, compared with the number of amino acids in the type I or type II Gγ subunits which were in the range 81–129, or 125–146 amino acids, respectively. These results resembled those previously reported from *Arabidopsis*, rice and soybean [11, 12, 19, 26].

Following the isolation and cloning of the first Gγ subunit gene from *Arabidopsis* [8], and with the development of high-throughput sequencing technology, bioinformatics and molecular biology techniques, the expression patterns and functional characteristics of Gγ subunits have been widely studied. For example, the type I Gγ subunit, represented by AGG1 in *Arabidopsis*, regulates lateral root formation by affecting the amount of the auxin 1-naphthaleneacetic acid (NAA) [2]. The type II Gγ subunit, represented by SlGGB1, regulates ABA and auxin signal transduction in tomato [10] whereas the type III Gγ subunit, represented by AGG3 in *Arabidopsis*, GmGγ8 in soybean and DEP1 and GS3 in rice, regulates lateral root development by affecting ABA signaling, organ size and number [21]. In order to analyze whether the Gγ subunit members of other Rosaceae species also have the same functional characteristics, the Gγ subunit gene expression patterns in Chinese white pear were investigated by qPCR. The results showed that Gγ subunit genes exhibited diverse spatiotemporal expression patterns in various organs, with *PbrGG4* being preferentially expressed in fruit, indicating that it may participate in fruit development. The expression pattern of this gene was similar to that of SlGGB1 in tomato, which was also mainly expressed in fruit, and has been implicated in the regulation of fruit morphogenesis through the auxin signaling pathway [10]. *PbrGG2, PbrGG3* and *PbrGG5*, from Chinese white pear, exhibited preferential expression in the root, implying that these genes might function in root growth under adverse conditions through the ABA signaling pathway. In *Arabidopsis*, AGG1 and AGG2 were mainly expressed in the root, and mediated the osmotic stress response and root formation and growth by the auxin and ABA signaling pathways [37, 40]. *PbrGG1* and *PbrGG7* were mainly expressed in the ovary, indicating that they were possibly involved in flower development and sexual reproduction in plants, a finding which is consistent with previous studies by northern blot analysis that AGG3 expression was highest in reproductive tissues [19]. *PbrGG3* exhibited high levels of expression in flower buds, which suggests a possible role in sexual reproduction in plants. This has not previously been reported for the G-protein subunits, and needs to be confirmed by experimentation.

Earlier studies have suggested that Gγ proteins are involved in a wide range of regulation of various abiotic stress and related signaling pathways affecting growth and development at the transcript level, and have a high potential for plant improvements [25, 26, 38]. For example, Yadav et al indicated that the expression of RGG1 and RGG2 genes in rice were dramatically induced by salinity and ABA treatment [24]. In soybean, Gγ subunit gene plays a key role in ABA-dependent signaling [27]. In rice, sun et al further demonstrated that the expression of Gγsubunit gene (*DEP1*) showed different nitrogen response, and the plants carrying the dominant dep1-1 allele exhibit increase nitrogen uptake and assimilation [41]. Here, we also analyzed the expression levels of Gγ subunit genes in pear by treating root and leaves with N-deficiency or ABA. The results showed that the expression levels of Gγ subunit genes were induced by abiotic stress, and therefor might play some specific functional in pear toward ABA-signaling pathway and nitrogen uptake and transport.

In addition, the subcellular localization analysis showed that subunits encoded by Gγ subunit genes from the three different clades, i.e., types I, II and III, were each located at the plasma membrane, with some also present in the nucleus or cytoplasm, indicating a potential capacity for Gγ subunit genes to act as signal transmitters on the plasma membrane at the subcellular level. In other species, similar subcellular localizations of the Gγ subunit proteins had been reported, with the Gγ subunits playing an important role in the correct binding of the G-protein on the plasma membrane to achieve its functional specificity [37]. In *Arabidopsis*, the AGG3-encoded protein was located on the plasma membrane, and shown to be involved in guard cell K^+^-channel regulation and morphological development [19]. The protein encoded by the Gγ subunit gene MGG1 was localized onto the plasma membrane, and it was suggested to act upstream of the cAMP signaling pathway, to play an important role in the regulation of conidiation, appressorium formation, mating and plant infection by the phytopathogenic fungus *M. oryzae* [31]. In rice, a series of assays confirmed the interaction of RGG1 with different stress-responsive proteins at the plasma membrane, playing crucial roles in signal transmission in response to various stresses to achieve tolerance [25].

## Conclusion

In summary, 35 Gγ subunit genes were identified and analyzed in eight Rosaceae species, with seven of these genes being from Chinese white pear. The gene structure, conserved protein motifs and phylogenetic tree analysis indicated that the Gγ subunit genes in the eight Rosaceae species were divided into three types (I, II and III). A WGD event strongly contributed to the expansion of the Gγ subunit gene family in species of the Maloideae subfamily, whereas singleton and dispersed duplication events played key roles in the expansion of the Gγ subunit gene family in species of the Prunoideae and Rosoideae subfamilies. Syntenic analysis showed that purifying selection was the primary evolutionary force imposed on Gγ subunit genes in the members of the Rosaceae family. Analysis of the relative expression of the seven Gγ subunit genes in Chinese white pear revealed that the expression of these genes was quite extensive across a range of organs, including near-universal expression in the fruit, root, ovary, and bud, moreover, the relative expression of *PbrGG* genes in pear root and leaf were induced by N-deficiency stress and ABA treatment, which suggests involvement in signal delivery, directly or indirectly, in these organs. Furthermore, subcellular localization showed that most of the Gγ subunits were located on the plasma membrane, suggesting a potential capacity for Gγ subunits to act as signal transmitters on plasma membranes at the subcellular level. These results provide useful information to achieve a better understanding of the evolution of the Gγ subunit gene family, and to form a foundation for further studies that will investigate the structure and function of the Gγ subunit gene family in the economically important plant family, the Rosaceae.

## Methods

### Identification of Gγ subunit genes in the Rosaceae

To identify the Gγ subunit genes in the Rosaceae, the Hidden Markov Model (HMM) profiles of the Gγ subunit domain (PF00631.22) were downloaded from the Pfam database (http://pfam.xfam.org/). Initially, the eight genome sequences were downloaded, namely the Chinese white pear (*Pyrus bretschneideri*, NJAU, v1.1) genome sequence from the Pear Genome Project of the Nanjing Agricultural University (NJAU) (http://peargenome.njau.edu.cn/), the strawberry (*Fragaria vesca*, GDR, v4.0), black raspberry (*Rubus occidentalis*, GDR, v3.0), European pear (*Pyrus communis*, GDR, v1.1), and sweet cherry (*Prunus avium*, GDR, v1.0) genome sequences from the Genome Database for the Rosaceae (GDR, http://www.Rosaceae.org/), the peach (*Prunus persica*, JGI, v2.1) and apple (*Malus domestica*, JGI, v1.1) genome sequences from the Joint Genome Institute (JGI, http://www.jgi.doe.gov/) and the Japanese apricot genome sequence from the *Prunus mume* (BFU v1.0) Genome Project (http://prunusmumegenome.bjfu.edu.cn/index.jsp). HMM searches were then performed against the local protein databases of the eight Rosaceae species using HMMER3 [42]. Additionally, BLAST algorithm-based searches were also performed against the eight Rosaceae genome databases, using full-length protein sequences of the identified Gγ subunit protein in *Arabidopsis*, and the candidate genes were selected with the e-values lower than 1e^−100^. A total of 58 putative Gγ subunit genes were screened from the eight Rosaceae species. Furthermore, all candidate Gγ subunit genes were manually verified with the Pfam (http://pfam.xfam.org) and SMART (http://smart.embl-heidelberg.de/) domain tools to confirm the presence and completeness of the GGL domain. Those amino acid sequences lacking the GGL domain or with redundant sequences were removed.

### Characterization of the Gγ subunit protein and phylogenetic analysis of the proteins in the Rosaceae

To identify and analyze the conserved motifs of the proteins encoded by the Gγ subunit gene family in the Rosaceae, a map of conserved motifs of all the Gγ subunit amino acid sequences was carried out with the Multiple Expectation Maximization for Motif Elicitation tool (http://meme-suite.org/tools/meme; MEME version 5.0.5), with the minimum and maximum motif width being six and 200, respectively, and the number of motifs being 15. The intron/exon structures of the Gγ subunit genes were determined by aligning the cDNA coding sequences with their corresponding genomic DNA sequences, which were displayed with the Gene Structure Display Server (http://gsds.cbi.pku.edu.cn/; GSDS 2.0). The protein isoelectric point (pI), formula, grand average of hydropathy (GRAVY), aliphatic index and molecular weight (MW) for all eight Rosaceae species were obtained from Expasy (https://web.expasy.org/protparam/). The protein length was acquired from the corresponding genome project of each species. Each of the Gγ subunit protein sequences from the eight Rosaceae species was aligned using the MUSCLE program. The phylogenetic tree was constructed and visualized using the Neighbor-Joining (NJ) method in MEGA6.0 software [43]. The parameters were set as follows: the p-distance and pairwise deletion option parameters were chosen, and the bootstrap tests was replicated 1,000 times.

### Chromosomal localization and syntenic analysis of the Gγ subunit gene family

The chromosomal locations of each of the Gγ subunit genes were provided in each download from the eight Rosaceae genome annotation databases. For syntenic analysis among the eight Rosaceae genomes, a method similar to that developed for the Plant Genome Duplication Database (PGGD) (http://chibba.agtec.uga.edu/duplication/) was conducted to determine the syntenic relationships of the Gγ subunit genes. Initially, BLASTP was conducted to search for all potential inter- and intra-pairwise blocks of Gγ subunit genes among the eight Rosaceae species. Subsequently, these homologous pairs were used as the input for the MCScanX toolkit to identify collinear chains [44]. MCScanX was further used to identify whole-genome duplication (WGD), tandem, dispersed, proximal duplication and transposed duplication events in the Gγ subunit gene family. Finally, the results were plotted as a circular graph using Circos software, though genes on unanchored scaffolds are not shown on the diagram [45].

### Calculation of Ka, Ks and Ka/Ks values of the Gγ subunit gene family

The values for synonymous substitutions (Ks) and non-synonymous substitutions (Ka) were acquired from the eight Rosaceae annotation files, using the MCScanX downstream analysis tools (Calculate_Ka_Ks_pipeline), and the Ka/Ks ratio values were calculated using these data. In brief, all inter- and intra-homologous gene pairs and the coding sequence were selected, on which were performed multiple alignments using computing_Ka_Ks_pipe.pl script by MAFFT software [46]. Then, those data were converted to AXT format for submission to the KaKs_Calculator in the GMYN model [47]. Finally, the readable data were generated, including Ka, Ks, and the *p*-value. The mean Ks was calculated by the six consecutive homologous gene pair on each side flanking the Gγ subunit genes. The divergence time was calculated with the formula T = Ks/2R, and R was taken to be 1.5×10^-8^ synonymous substitutions per site per year for dicotyledonous [48].

### RNA extraction and qPCR assays for *PbrGG* genes

To analyze the expression patterns of Gγ subunit genes in Chinese white pear, total RNA was extracted independently from different pear tissues using an RNA kit (RNAsimple Total RNA Kit; Tiangen, Beijing, China), according to the manufacturer’s instructions. Of the tissues used, were picked from the pear germplasm orchard of the Center of Pear Engineering Technology Research located at Hushu in Nanjing. The petal, sepal, ovary, ripening fruit and pollen grain tissue were harvested from a 6-year-old Chinese white pear tree (‘Dangshan suli’), and pollen tubes were obtained from pollen grains cultured for 5 h, whereas root, stem and leaf tissue were harvested from 2-year-old Chinese white pear seedlings (‘Dangshan suli’). For the expression levels of Gγ subunit genes in response to N-deficiency, 5-week-old hydroponic pear seedling were transferred into the nutrient solution of N-deficiency for five days, and samples were harvested from roots. For the expression levels of Gγ subunit genes in response to ABA treatment, the leaves of 3-month-old pear seedlings were sprayed with 50 μM ABA solutions, and samples were harvested from leaves at 6 h after treatment. Total RNA was reverse transcribed using the M-MLV reverse transcriptase (Takara Bio, Shanghai, China). The specific primers for the Gγ subunit genes of Chinese white pear were designed using the Primer Premier 5.0 software [35], *PbrActin* was selected as the internal standard housekeeping gene and all primers are shown in Table S5. The total qPCR reaction volume was 20 μL, consisting of 4.9 μL ddH_2_O, 10 μL of 2 × SYBR Green Master Mix, 0.1 μL (100 ng) of cDNA, and 5 μL (200 nM) of the gene-specific primer mixture. The qPCR was performed using LightCycler SYBR Green I Master (Roche, USA), and the procedure was as follows: pre-incubation at 95 °C for 5 min, then 45 cycles at 95 °C for 3 s, 60 °C for 10 s and 72 °C for 30 s, and a final extension at 72 °C for 3 min. Relative (to the internal standard) expression levels were calculated by LightCycler 480 Software v.1.5.0 (Roche, USA), using the 2^−ΔΔCt^ method [36]. Three biological and three technical replicates were performed for each experiment, and the data were analyzed by SPSS17.0 (IBM, Armonk, NY, USA) and visualized by GraphPad Prism 8 (GraphPad Software, San Diego, CA, USA) [49].

### Subcellular localization assays

The full-length coding sequence (CDS) of the Gγ subunit genes *PbrGG1*, *PbrGG4* and *PbrGG6* were amplified from Chinese white pear root, leaf and fruit by PCR, using the corresponding primers, which are listed in Table S5. The amplified PCR products were cloned into the modified *pCAMBIA1300-GFP* vector carrying the CaMV 35s promoter (Clontech, Beijing, China). Subsequently, the fusion plasmids 35s::*PbrGG*-*GFP* and the control plasmid 35s:*GFP* were independently transferred into *Agrobacterium tumefaciens* cells by electroporation. *Agrobacterium* cells transformed with the respective fusion plasmid were then injected into tobacco (*Nicotiana benthamiana*) leaves, and the green fluorescence signals were visualized with a Zeiss LSM 780 Image Browser (Carl Zeiss, Inc., Thornwood, NY, USA) 3 d after transformation. Three independent experiments were performed for each gene.

## Supplementary Information

**Additional file 1: Table S1**. Numbers of Gγ subunit genes from different origins in eight Roseceae genomes

**Additional file 2: Table S2.** Synteny analysis of Gγ subunit gene regions in Rosaceae

**Additional file 3: Table S3.** Ka, Ks, Ka/Ks, and divergence-time of paralogous genes among Gγ subunit gene family in Rosaceae

**Additional file 4: Table S4.** Prediction of subcellular localization of Gγ subunit protein in Chinese white pear

**Additional file 5.** **Table S5.** List of primers used in this work

## Data Availability

The data and material that support the findings of this study are available from the corresponding author on request. The pear genome datasets used during the current study are available in our pear center website (http://peargenome.njau.edu.cn/); the strawberry, black raspberry, European pear, sweet cherry genome sequences were downloaded from the Genome Database for the Rosaceae (http://www.Rosaceae.org/); The sequence of apple and peach were downloaded from the Joint Genome Institute (http://www.jgi.doe.gov/); the Japanese apricot genome sequence were downloaded from the *Prunus mume* Genome Project (http://prunusmumegenome.bjfu.edu.cn/index.jsp). The RNA-seq data were obtained from the NCBI database (https://www.ncbi.nlm.nih.gov/).

## Abbreviations

Gγ subunit: Gamma subunit of heterotrimeric GTP binding protein
PGGD: Plant Genome Duplication Database
WGD: Whole-genome duplication
HMM: Hidden Markov Model
Ks: Synonymous substitutions
Ka: Non-synonymous substitutions
MEME: Multiple Expectation Maximization for Motif Elicitation
qRT-PCR: Quantitative real-time polymerase chain reaction
GFP: Green Fluorescent Protein
